# Characterization of non-adopters of COVID-19 non-pharmaceutical interventions through a national cross-sectional survey to assess attitudes and behaviours

**DOI:** 10.1038/s41598-021-01279-2

**Published:** 2021-11-05

**Authors:** Raynell Lang, Omid Atabati, Robert J. Oxoby, Mehdi Mourali, Blake Shaffer, Hasan Sheikh, Madison M. Fullerton, Theresa Tang, Jeanna Parsons Leigh, Braden J. Manns, Deborah A. Marshall, Noah M. Ivers, Scott C. Ratzan, Jia Hu, Jamie L. Benham

**Affiliations:** 1 Department of Medicine, Cumming School of Medicine, University of Calgary, Calgary, AB Canada; 2 Department of Economics, Faculty of Arts, University of Calgary, Calgary, AB Canada; 3 Haskayne School of Business, University of Calgary, Calgary, AB Canada; 4 Department of Family and Community Medicine, University of Toronto, Toronto, ON Canada; 5 Department of Community Health Sciences, Cumming School of Medicine, University of Calgary, Calgary, AB Canada; 6 Faculty of Health, School of Health Administration, Dalhousie University, Halifax, NS Canada; 7 Department of Critical Care Medicine, University of Calgary, Calgary, AB Canada; 8 Women’s College Hospital Institute for Health System Solutions and Virtual Care, Women’s College Hospital, Toronto, ON Canada; 9 Institute for Health Policy, Management and Evaluation, University of Toronto, Toronto, ON Canada; 10 City University of New York Graduate School of Public Health & Health Policy, New York, NY USA

**Keywords:** Public health, Epidemiology, Infectious diseases, Viral infection

## Abstract

Adoption of non-pharmaceutical interventions (NPIs) remains critical to curtail the spread of COVID-19. Using self-reported adherence to NPIs in Canada, assessed through a national cross-sectional survey of 4498 respondents, we aimed to identify and characterize non-adopters of NPIs, evaluating their attitudes and behaviours to understand barriers and facilitators of adoption. A cluster analysis was used to group adopters separately from non-adopters of NPIs. Associations with sociodemographic factors, attitudes towards COVID-19 and the public health response were assessed using logistic regression models comparing non-adopters to adopters. Of the 4498 respondents, 994 (22%) were clustered as non-adopters. Sociodemographic factors significantly associated with the non-adoption cluster were: (1) being male, (2) age 18–34 years, (3) Albertans, (4) lower education level and (5) higher conservative political leaning. Participants who expressed low concern for COVID-19 and distrust towards several institutions had greater odds of being non-adopters. This information characterizes individuals at greatest odds for non-adoption of NPIs to inform targeted marketing interventions.

## Introduction

Over 1 year following the discovery of SARS-CoV-2 and with over 194 million cases and 4.1 million deaths globally as of July 2021^1^, the COVID-19 pandemic has emerged as one of the greatest public health crises of the last century. The negative impacts of the COVID-19 pandemic have been extensive and broad reaching impacting healthcare, the economy and the general population globally^2–6^. In an effort to curtail the spread of SARS-CoV-2, medical and public health communities have worked together with governments to provide recommendations and develop policies for promoting uptake of public health behaviors (now commonly referred to as non-pharmaceutical interventions (NPIs)), including limiting social gatherings, physical distancing, using face masks, avoiding public spaces and quarantining when sick^7–9^.

The implementation of NPIs has coincided with a decline in the number of new COVID-19 cases among many different countries^8,10–12^. Using modelling, the number of cases in mainland China at the end of February 2020 were estimated to be 67-fold higher without NPIs than with NPIs^8^. A model was also used to estimate the effect of NPIs across 11 European countries between February and May 2020. This model estimated that the interventions were sufficient to drive the time-varying reproduction number below one and demonstrated that NPIs had a large effect on reducing transmission of COVID-19^10^. Unfortunately, these NPIs have also led to high economic, health and social costs globally^13–16^.

Distinct attitudes towards NPIs for COVID-19 have emerged with variation in the public’s agreement with and willingness to adopt these recommendations^9,17–20^. Prior studies have demonstrated higher adoption for NPIs among individuals who have higher concern about the harms of SARS-CoV-2, a greater knowledge of the pandemic, are older ($\ge$ 50 years), are female and are highly educated^17,20^. Willingness to adopt NPIs during both the COVID-19 pandemic and prior disease outbreaks have also been linked to the level of trust people have in government and institutions^21,22^. Misinformation and COVID-19 related conspiracy theories contribute towards non-adoption of NPIs^23,24^. Usage of traditional news media (i.e., print, television or radio) has been typically associated with greater adherence and less conspiracy beliefs compared to people who use social media as an information source^23^.

With the recent approval of several COVID-19 vaccinations across the globe^25^, and the initiation of a mass roll-out strategy, many individuals are under false assumptions that NPIs are no longer needed or important in controlling the spread of COVID-19. However, experts are warning that as vaccinations are distributed, the public must continue to adhere to NPIs^26,27^. Public health messaging will be critical during vaccine roll-out to encourage adoption and continued adherence to NPIs^26^. Lack of information transparency and failure to customize information to different subpopulations have been cited as major factors contributing to communication ineffectiveness regarding emerging infectious diseases in the past and the COVID-19 pandemic thus far^17,28–30^. Therefore, identifying the characteristics of individuals who are most likely to be non-adopters is crucial to the development of effective communication strategies including; knowledge translation tools, targeted marketing programs, and community engagement.

We aimed to characterize self-reported non-adopters of NPIs compared to people who reported following these behaviors. We wanted to understand the differences in sociodemographic factors, attitudes towards COVID-19 and risk and trust measures across these 2 groups. We also wanted to identify the informational and social media platforms they use to obtain their COVID-19 data. This information will help identify people at highest risk for non-adoption of NPIs to inform targeted marketing interventions, including optimal communication strategies and platforms, to encourage behaviour change among non-adopters and reduce spread of COVID-19.

## Results

**Figure 1:**
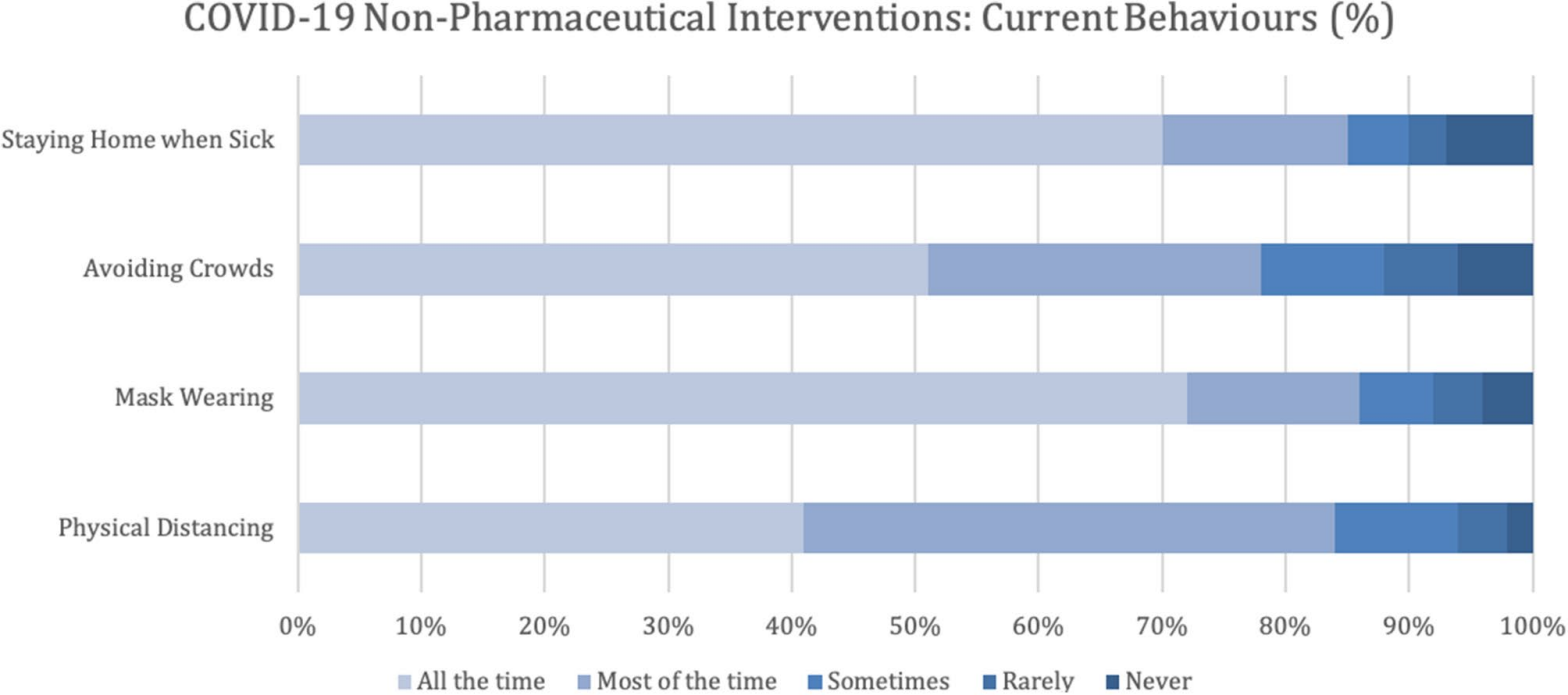
Self-reported adherence to non-pharmaceutical interventions for COVID-19, based on the question; over the last few weeks how often have you been performing each of the listed behaviours? (n = 4498).

The online survey was distributed to 14,887 participants and 5893 respondents (40%) clicked on the survey link, with 1395 (24% of respondents) excluded due to not completing and submitting the survey. A response rate of 40% is typical for the Angus Reid Forum^31^ as they distribute broadly to their panel to promote quick responses. The proportion of individuals who completed the survey were similar in age, sex, ethnicity, highest level of education, and province of residence to those who did not complete the survey. Overall, 4498 participants completed the survey (Table 1) and were included in the analysis. The majority of participants were female (2298, 51%), Caucasian (3866, 85%) and educated with either an undergraduate or graduate university degree (1314, 29%). There were 1998 (44%) respondents from Alberta. The age of participants ranged from 18 to 94 years old, with a mean of 47 years ($\pm$ 16 years). For information on sociodemographic factors associated with each NPI please see Supplemental Table S1. There were 721 (16%) participants who reported that over the last few weeks they maintained physical distancing sometimes/rarely or never, 631 (14%) who masked sometimes/rarely or never, 986 (22%) who avoided crowded spaces sometimes/rarely or never and 646 (14%) who stayed home while sick, sometimes/rarely or never (Fig. 1).

**Table 1:** Sociodemographic factors associated with adoption clusters for COVID-19 non-pharmaceutical interventions.

Characteristic	Total (N = 4489) (%)	Adopter cluster (N = 3504) (%)	Non-adopter cluster (N = 994) (%)	P-value	cOR	95% CI	aOR	95% CI
**Biologic sex**								
Male (REF)	2204 (49)	1571 (71)	633 (29)	< 0.001	1.00	–	1.00	–
Female	2294 (51)	1933 (84)	361 (16)		0.46	0.40–0.54	0.55	0.47–0.65
**Age (years)**								
18–34 (REF)	1341 (30)	1009 (75)	332 (25)	< 0.001	1.00		1.00	–
35–54	1585 (35)	1169 (74)	416 (26)		1.08	0.92–1.28	0.64	0.53–0.79
$$\ge$$ 55	1572 (35)	1326 (84)	246 (16)		0.56	0.47–0.68	0.25	0.20–0.31
**Province**								
Alberta (REF)	1998 (44)	1407 (70)	591 (30)	< 0.001	1.00	–	1.00	–
British Columbia	502 (11)	429 (85)	73 (15)		0.41	0.31–0.53	0.56	0.42–0.76
Prairie Provinces^+^	445 (10)	349 (78)	96 (22)		0.65	0.51–0.84	0.55	0.42–0.73
Ontario	800 (18)	686 (86)	114 (14)		0.40	0.32–0.49	0.49	0.39–0.63
Quebec	502 (11)	418 (83)	84 (17)		0.48	0.37–0.62	0.83	0.62–1.11
Atlantic Provinces^+^	251 (6)	251 (86)	36 (14)		0.40	0.28–0.57	0.57	0.38–0.86
**Annual household income**								
< $50,000 (REF)	951 (21)	774 (81)	177 (19)	0.003	1.00	–	1.00	–
$50,000-$99,999	1332 (30)	1048 (79)	284 (21)		1.19	0.96–1.46	0.97	0.76–1.24
$100,000-$199,999	1350 (30)	1041 (77)	309 (23)		1.30	1.06–1.60	0.96	0.75–1.23
$$\ge$$$200,000	214 (5)	153 (72)	61 (29)		1.74	1.24–2.45	0.99	0.66–1.50
Rather not say	651 (14)	488 (75)	163 (25)		1.46	1.15–1.86	1.13	0.86–1.50
**Highest education**								
High school graduate or less (REF)	897 (20)	647 (72)	250 (28)	< 0.001	1.00	–	1.00	–
Some college or trade school	840 (19)	621 (74)	219 (26)		0.91	0.74–1.13	0.99	0.78–1.28
College or trade school	996 (22)	743 (75)	253 (25)		0.88	0.72–1.08	0.86	0.68–1.09
Some University	454 (10)	351 (77)	103 (23)		0.76	0.58–0.99	1.01	0.74–1.37
University degree	1311 (29)	1142 (87)	169 (13)		0.38	0.31–0.48	0.49	0.37–0.63
**Race/ethnicity**								
Caucasian (REF)	3862 (86)	3002 (78)	860 (22)	0.006	1.00	–	1.00	–
Indigenous/First Nations/Metis/Inuit	228 (5)	169 (74)	59 (26)		1.22	0.90–1.65	1.07	0.75–1.53
Chinese/Filipino/Other Asian	124 (3)	108 (87)	16 (13)		0.52	0.30–0.88	0.55	0.30–1.00
Caribbean/South American/African	70 (2)	57 (81)	13 (19)		0.80	0.43–1.46	0.96	0.49–1.88
Middle Eastern/Central Asian/South Asian	69 (2)	62 (90)	7 (10)		0.39	0.18–0.86	0.50	0.21–1.17
Other	146 (3)	106 (73)	39 (27)		1.28	0.88–1.87	0.98	0.63–1.51
**Political leaning**								
Very liberal	603 (13)	573 (95)	30 (5)	< 0.001	0.26	0.17–0.39	0.23	0.15–0.35
Liberal	805 (18)	746 (93)	59 (7)		0.39	0.29–0.54	0.42	0.30–0.58
Slightly liberal	433 (10)	399 (92)	34 (8)		0.42	0.29–0.62	0.42	0.28–0.62
Moderate/middle of the road (REF)	1029 (23)	857 (83)	172 (17)		1.00	–	1.00	–
Slightly conservative	485 (11)	340 (70)	145 (30)		2.12	1.65–2.74	2.02	1.54–2.63
Conservative	807 (18)	450 (56)	357 (44)		3.95	3.19–4.48	4.12	3.26–5.21
Very conservative	336 (7)	139 (41)	197 (59)		7.06	5.38–9.27	7.00	5.19–9.41

Odds ratios are the odds of being in the non-adopter cluster compared to odds of being in the adopter cluster.

*CI* confidence interval, *REF* reference group, *cOR* crude odds ratio using logistic regression, *aOR* adjusted odds ratio, adjusted for sex, age, province of residence, annual household income, highest education, political leaning and ethnicity.

^+^Prairie provinces included Saskatchewan and Manitoba; Atlantic provinces include Nova Scotia, New Brunswick, Prince Edward Island and Newfoundland and Labrador.

### Adopter cluster vs non-adopter cluster sociodemographic factors

Using a cluster analysis, two distinct non-overlapping clusters were identified: 3504 (78%) were clustered as adopters and 994 (22%) were non-adopters (Supplemental Table S2). Adoption of NPIs differed by age, sex, province of residence, race/ethnicity, annual income, education level, and political leaning (Table 1). A multivariable model, adjusted for all sociodemographic variables identified that annual household income and ethnicity was not associated with differences between adoption clusters and therefore not used in the subsequent adjusted analyses. Compared to those aged 18–34 years, participants age 35–54 years (aOR 0.64, 95% CI 0.53–0.79) and $\ge$ 55 years (aOR 0.25, 95% CI 0.20–0.31) and those of female sex (aOR 0.55, 95% CI 0.47–0.65) had lower odds of being in the non-adopter cluster. Albertans had greater odds of being non-adopters compared with all other provinces; however, when adjusted, this association was no longer significant for Quebec participants. Education was negatively associated with non-adoption, with respondents having a university degree (aOR 0.49, 95% CI 0.37–0.63) having lower odds of being non-adopters than respondents who graduated high school or less. People of Middle Eastern/Central Asian/South Asian (OR 0.39, 95% CI 0.18–0.86) and Chinese/Filipino/Other Asian (OR 0.52, 95% CI 0.30–0.88) ethnicity had lower odds of non-adoption compared with Caucasians; however, this association attenuated following adjustment for other demographics.

People reporting slightly liberal (aOR 0.42, 95% CI 0.28–0.62), liberal (OR 0.42, 95% CI 0.30–0.58) or very liberal (aOR 0.23, 95% CI 0.15–0.35) political leaning had lower odds of non-adoption compared to people reporting moderate/middle of the road political leaning. Conversely, compared with moderate/middle of the road, people reporting slightly conservative (aOR 2.02, 95% CI 1.54–2.63), conservative (aOR 4.12 95% CI 3.26–5.21) or very conservative (aOR 7.00, 95% CI 5.19–9.41) political views had greater odds of non-adoption (Table 1).

### Attitudes towards COVID-19 between adopter and non-adopter clusters

Following adjustment for demographic variables including sex, age, province of residence, highest education level and political leaning, concern for oneself and for others of becoming infected with COVID-19 were significantly associated with adoption of NPIs. The adjusted odds of non-adoption for respondents who reported being not at all concerned about their friends or family getting sick were 31 times (95% CI 21.77–41.16) higher than respondents expressing concern about their friends or family becoming sick (Table 2). Participants who felt that they would have mild or no symptoms if they were to contract COVID-19 had increased odds (aOR 3.52, 95% CI 2.91–4.26) for non-adoption than people who felt that their symptoms would be manageable. Having known someone with COVID-19 or living with someone they considered high risk for severe outcome of COVID-19 were positively associated with adoption. Non-adopters were more likely to interact with more people outside their household, with 37% reporting having regularly interacted with more than 20 people over the past few weeks compared with 12% of adopters (Table 2).

**Table 2:** Associations between attitudes and behaviours regarding COVID-19 and adoption clusters for COVID-19 NPIs.

Attitudes and behaviours towards COVID-19	Odds of being a non-adopter compared to adopter						
	Total N = 4498 (%)	Adopter Cluster (N = 3504) (%)	Non-Adopter Cluster (N = 994) (%)	OR	95% CI	aOR	95% CI
**Ever tested positive for COVID-19**							
No (REF)	4385 (97)	3413 (78)	972 (22)	1.00	–	1.00	–
Yes	113 (3)	91 (81)	22 (19)	0.85	0.53–1.36	0.57	0.52–1.52
**Know someone who had COVID-19**							
No (REF)	3162 (70)	2401 (76)	761 (24)	1.00	–	1.00	–
Yes	1336 (30)	1103 (83)	223 (17)	**0.32**	**0.57–0.78**	**0.71**	**0.48–0.67**
**Extent to which COVID-19 might affect your health**							
Mild or no symptoms	1085 (24)	536 (49)	549 (51)	**4.96**	**4.19–5.87**	**3.52**	**2.91–4.26**
Manageable (REF)	1940 (43)	1608 (83)	332 (17)	1.00	**–**	1.00	**–**
Severe symptoms	1026 (23)	945 (92)	81 (8)	**0.42**	**0.32–0.54**	**0.51**	**0.39–0.67**
Possible death	447 (10)	415 (93)	32 (7)	**0.37**	**0.26–0.55**	**0.46**	**0.30–0.69**
**Level of concern about friends/family getting sick**							
Very concerned	1439 (32)	1384 (96)	55 (4)	**0.33**	**0.24–0.44**	**0.38**	**0.29–0.52**
Concerned (REF)	1855 (41)	1654 (89)	201 (11)	1.00	**–**	1.00	**–**
Not that concerned	834 (19)	414 (50)	420 (50)	**8.35**	**6.84–10.19**	**6.10**	**4.90–7.59**
Not at all concerned	370 (8)	52 (14)	318 (86)	**50.32**	**36.26–69.84**	**31.01**	**21.77–41.16**
**Live with someone who is high risk**							
No (REF)	2649 (59)	1970 (74)	679 (26)	1.00	**–**	1.00	**–**
Yes	1849 (41)	1534 (83)	315 (17)	**0.60**	**0.51–0.69**	**0.69**	**0.58–0.82**
**Number of persons outside your household that you regularly interact with in last few weeks**							
1–5 persons (REF)	1934 (43)	1743 (90)	191 (10)	1.00	**–**	1.00	**–**
6–10 persons	1048 (23)	859 (82)	189 (18)	**2.00**	**1.62–2.49**	**1.75**	**1.37–2.23**
11–15 persons	485 (11)	334 (69)	151 (31)	**4.13**	**3.23–5.26**	**3.12**	**2.38–4.12**
16–20 persons	257 (6)	157 (61)	100 (39)	**5.81**	**4.34–7.78**	**3.94**	**2.81–5.51**
> 20 persons	774 (17)	411 (53)	363 (47)	**8.06**	**6.56–9.90**	**5.96**	**4.71–7.53**
**Younger people are mostly to blame for the increase in cases**							
Strongly agree/agree (REF)	1968 (44)	1751 (89)	217 (11)	1.00	**–**	1.00	**–**
Strongly disagree/disagree	1881 (42)	1256 (67)	625 (33)	**4.02**	**3.39–4.76**	**4.29**	**3.53–5.21**
Unsure/can’t say	649 (14)	497 (77)	152 (23)	**2.47**	**1.96–3.11**	**2.48**	**1.91–3.22**
**There is too much focus on how COVID-19 affects older people**							
Strongly agree/agree (REF)	1228 (27)	883 (72)	345 (28)	1.00	**–**	1.00	**–**
Strongly disagree/disagree	2942 (65)	2415 (82)	527 (18)	**0.56**	**0.48–0.65**	**0.63**	**0.52–0.75**
Unsure/can’t say	328 (7)	206 (63)	122 (37)	**1.52**	**1.17–1.96**	1.22	0.90–1.65

Bold signals statistically significant p-values (< 0.05).

Odds ratios are the odds of being in the non-adopter cluster compared to odds of being in the adopter cluster.

*CI* confidence interval, *REF* reference group, *cOR* crude odds ratio using logistic regression, *aOR* adjusted odds ratio, adjusted for sex, age, province of residence, highest education and political leaning.

Respondents who disagreed with the statement that “younger people are mostly to blame for the increase in cases” had greater odds of being non-adopters of NPIs (aOR 4.29, 95% CI 3.53–5.21); however, 36% of people who disagreed with this statement were of the age 18–34 years compared to 27% of people $\ge$ 55 years old. Therefore, the youngest age group were more likely to disagree that younger people were to blame for the increase in cases, yet had higher odds of being in the non-adopter cluster.

### Attitudes towards NPIs and reasons for non-adoption

Adoption of NPIs were positively correlated with adoption of contact tracing and exposure notification apps, with respondents who had downloaded an app having lower odds of being non-adopters (aOR 0.30, 95% CI 0.24–0.38) than those who had not downloaded an app (Table 3). The odds of being in the non-adopter cluster was greater among people who disagreed with the statement that public health messaging had been clear and understandable (aOR 3.69, 95% CI 3.09–4.40) compared with those who agreed. There was also a positive association between non-adoption and disagreement that public health messaging had been consistent (aOR 3.69, 95% CI 3.06–4.45). The majority of respondents agreed that COVID-19 restrictions were harming the economy (3024, 77%), however people who disagreed with this comment had lower odds of non-adoption (aOR 0.43, 95% CI 0.32–0.59) compared with people who agreed.

**Table 3:** Associations between attitudes towards NPIs and public health messaging and adoption clusters for COVID-19 NPIs.

Attitudes towards COVID-19 public health behaviours and messaging	Odds of being a non-adopter compared to adopter						
	Total N = 4498 (%)	Adopter cluster (N = 3504) (%)	Non-adopter cluster (N = 994) (%)	OR	95% CI	aOR	95% CI
**Have you downloaded a contact tracing application for COVID-19**							
No (REF)	3105 (69)	2211 (71)	894 (29)	1.00	–	1.00	–
Yes	1393 (31)	1293 (93)	100 (7)	**0.19**	**0.15–0.24**	**0.30**	**0.24–0.38**
**Public health messaging has been clear/understandable**							
Strongly agree/Agree (REF)	2548 (57)	2274 (89)	274 (11)	1.00	–	1.00	–
Strongly disagree/disagree	1835 (41)	1155 (63)	680 (37)	**4.89**	**4.18–5.72**	**3.69**	**3.09–4.40**
Unsure/can’t say	115 (3)	75 (65)	40 (35)	**4.43**	**2.96–6.63**	**3.45**	**2.14–5.56**
**Public health messaging around COVID-19 has been consistent**							
Strongly agree/agree (REF)	2195 (49)	1974 (90)	221 (10)	1.00	–	1.00	–
Strongly disagree/disagree	2155 (48)	1438 (67)	717 (33)	**4.45**	**3.77–5.25**	**3.69**	**3.06–4.45**
Unsure/can’t say	148 (3)	92 (62)	56 (38)	**5.44**	**3.79–7.79**	**4.69**	**3.09–7.10**
**COVID-19 restrictions are harming our economy**							
Strongly agree/agree (REF)	3477 (77)	2555 (73)	922 (27)	1.00	–	1.00	–
Strongly disagree/disagree	838 (19)	781 (93)	57 (7)	**0.20**	**0.15–0.27**	**0.43**	**0.32–0.59**
Unsure/can’t say	183 (4)	168 (92)	15 (8)	**0.25**	**0.15–0.42**	**0.42**	**0.24–0.74**
**Has messaging/advertising had an impact on your likelihood to do these?**							
Physical distancing							
Much more/more likely	3024 (67)	2793 (92)	231 (8)	**0.11**	**0.09–0.13**	**0.13**	**0.11–0.16**
No difference (REF)	1173 (26)	667 (57)	506 (43)	1.00	–	1.00	–
Lot less/little less likely	239 (5)	27 (11)	212 (89)	**10.35**	**6.82–15.70**	**8.34**	**5.24–13.28**
Not seen any messaging or advertising	62 (1)	17 (27)	45 (73)	**3.49**	**1.97–6.17**	**10.48**	**5.40–20.34**
Masking							
Much more/more likely	3108 (69)	2902 (93)	206 (7)	**0.10**	**0.08–0.12**	**0.13**	**0.11–0.17**
No difference (REF)	960 (21)	559 (58)	401 (42)	1.00	–	1.00	–
Lot less/little less likely	388 (9)	38 (10)	350 (90)	**12.84**	**8.97–18.38**	**9.49**	**6.44–13.99**
Not seen any messaging or advertising	42 (1)	5 (12)	37 (88)	**10.32**	**4.02–26.48**	**35.81**	**12.98–98.82**
Avoiding public places							
Much more/more likely	3033 (67)	2846 (94)	187 (6)	**0.72**	**0.06–0.09**	**0.09**	**0.07–0.11**
No difference (REF)	1138 (25)	596 (52)	542 (48)	1.00	–	1.00	–
Lot less/little less likely	269 (6)	44 (16)	225 (84)	**5.62**	**3.99–7.93**	**4.34**	**2.96–6.36**
Not seen any messaging or advertising	58 (1)	18 (31)	40 (69)	**2.44**	**1.38–4.31**	**7.29**	**3.84–13.86**
Staying home when sick							
Much more/more likely	3203 (71)	2845 (89)	358 (11)	**0.16**	**0.13–0.19**	**0.17**	**0.14–0.20**
No difference (REF)	1131 (25)	631 (56)	500 (44)	1.00	–	1.00	–
Lot less/little less likely	107 (2)	22 (21)	85 (79)	**4.88**	**3.01–7.91**	**5.08**	**2.87–8.98**
Not seen any messaging or advertising	57 (1)	6 (11)	51 (89)	**10.73**	**4.57–25.20**	**36.90**	**14.52–93.72**

Bold signals statistically significant p-values (< 0.05).

Odds ratios are the odds of being in the non-adopter cluster compared to odds of being in the adopter cluster.

*CI* confidence interval, *REF* reference group, *cOR* crude odds ratio using logistic regression, *aOR* adjusted odds ratio, adjusted for sex, age, province of residence, highest education and political leaning.

Individuals who responded that messaging or advertising made them less likely to physical distance (aOR 8.34, 95% CI 5.24–13.28), mask in indoor public spaces (aOR 9.49, 95% CI 6.44–13.99), avoid public spaces (aOR 4.34, 95% CI 2.96–6.36) and stay home when sick (OR 5.08, 95% CI 2.87–8.98) had significantly higher odds of non-adoption compared to people reporting no difference in their behaviors with messaging or advertising. Across all NPIs evaluated, people who reported not seeing any messaging or advertising had significantly higher odds of non-adoption (Table 3). When evaluating messaging for physical distancing, 62 (1.4%) people reported they had not seen any messaging. While this is a low percentage, it represents a key target population that communication efforts had missed. Of these individuals, 58% were female, 48% from Alberta, 83% Caucasian race/ethnicity and 84% were aged 18–55 years old. These demographic proportions were similar for people reporting not seeing messaging across each evaluated NPI.

**Figure 2:**
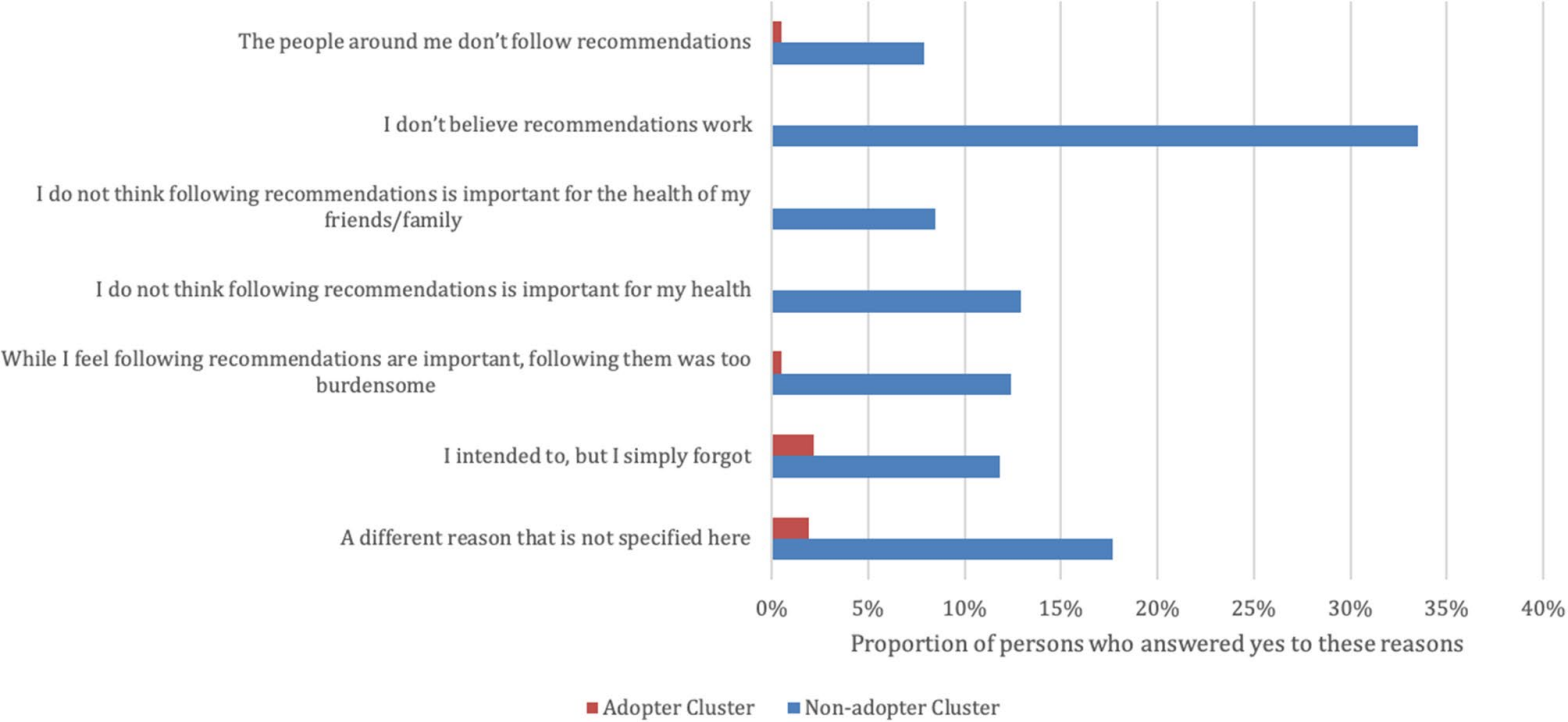
Self -reported reasons for having not followed the public health recommendations over the past few weeks by adoption clusters (n = 4498).

The greatest proportion of respondents in the non-adopter cluster reported that they did not believe that the recommendations work as the reason for having not followed the public health guidelines (333/994, 34%). Whereas the greatest proportion of adopters reported that they had intended to follow the guideline but simply forgot (78/3504, 2%) as the reason for having not followed public health guidelines (Fig. 2). Other main reasons non-adopters reported not following NPIs included: they did not think the recommendations were important for their health (129/994, 13%) or the health of their friends and family (85/994, 9%), or the NPIs were too burdensome to follow (124/994, 12%). Fewer reported their behaviours were influenced by those around them not following recommendations (79/994, 8%) (Fig. 2).

### Trust in institutions

Distrust across all institutions was associated with non-adoption of NPIs (Table 4). The most predictive trust factor was trust in government, with people who reported trusting government (1404, 31%) having lower odds (aOR 0.43, 95% CI 0.32–0.57) of being in the non-adopter cluster adjusted for sex, age, providence of residence, highest education and political leaning compared to people who reported neither trusting nor distrusting government. Whereas people who reported distrust in government (1685, 37%) had higher odds of being in the non-adopter cluster (aOR 3.71, 95% CI 3.05–4.52) compared to respondents who reported neither trusting nor distrusting government. People who had trust in healthcare (3373, 75%) also had lower odds of being in the non-adoption cluster (aOR 0.26, 95% CI 0.21–0.31) and those that distrusted healthcare had a higher odds of being in the non-adoption cluster (aOR 2.28, 95% CI 1.67–3.10) compared to people reporting neither trusting nor distrusting healthcare. Distrust in technology, financial and professional service institutions were also associated with reduced adoption of NPIs. Participants who expressed high trust in retail (1205, 27%) had greater odds of non-adoption for physical distancing and avoiding public spaces compared to people who reported neither trusting/nor distrusting retail (Table 4).

**Table 4:** Association with trust in specific institutions with adoption of COVID-19 non-pharmaceutical interventions.

Trust in specific institutions	Odds of not being compliant with the COVID-19 non-pharmaceutical intervention								
	Total (%)	Physical distancing		Masking		Avoiding public spaces		Staying home when sick	
		OR	95% CI	OR	95% CI	OR	95% CI	OR	95% CI
**Technology**									
Completely trust/trust	580 (13)	0.78	0.56–1.07	0.80	0.57–1.12	0.83	0.64–1.08	**0.71**	**0.52–0.97**
Neither trust nor distrust (REF)	1709 (38)	1.00		1.00		1.00	–	1.00	–
Completely distrust/distrust	2214 (49)	**2.09**	**1.75–2.50**	**2.14**	**1.77–2.60**	**1.63**	**1.40–1.91**	**1.31**	**1.10–1.57**
**Financial**									
Completely trust/trust	1326 (29)	0.83	0.68–1.02	**0.77**	**0.63–0.96**	0.91	0.76–1.08	**0.80**	**0.65–0.99**
Neither trust nor distrust (REF)	1825 (41)	1.00		1.00		1.00	–	1.00	–
Completely distrust/distrust	1352 (30)	**1.40**	**1.16–1.68**	1.12	0.92–1.36	**1.25**	**1.06–1.48**	1.16	0.95–1.40
**Retail**									
Completely trust/trust	1205 (27)	**1.21**	**1.01–1.47**	1.20	0.99–1.46	**1.28**	**1.09–1.52**	1.06	0.87–1.29
Neither trust nor distrust (REF)	2142 (48)	1.00		1.00		1.00	–	1.00	–
Completely distrust/distrust	1156 (26)	1.02	0.84–1.24	0.92	0.74–1.13	0.98	0.82–1.16	0.95	0.77–1.16
**Professional services**									
Completely trust/trust	2293 (51)	**0.82**	**0.69–0.98**	0.84	0.70–1.01	0.95	0.82–1.11	0.93	0.77–1.12
Neither trust nor distrust (REF)	1681 (37)	1.00		1.00		1.00	–	1.00	–
Completely distrust/distrust	529 (12)	**1.59**	**1.25–2.02**	**1.44**	**1.11–1.85**	**1.35**	**1.08–1.69**	**1.47**	**1.14–1.89**
**Healthcare**									
Completely trust/trust	3373 (75)	**0.33**	**0.27–0.39**	**0.26**	**0.21–0.31**	**0.37**	**0.32–0.44**	**0.45**	**0.37–0.55**
Neither trust nor distrust (REF)	830 (18)	1.00		1.00		1.00	–	1.00	–
Completely distrust/distrust	300 (7)	**2.02**	**1.53–2.65**	**2.02**	**1.53–2.66**	**1.72**	**1.32–2.25**	**1.55**	**1.15–2.08**
**Government**									
Completely trust/trust	1404 (31)	**0.49**	**0.36–0.65**	**0.31**	**0.21–0.45**	**0.57**	**0.46–0.72**	**0.75**	**0.58–0.96**
Neither trust nor distrust (REF)	1414 (31)	1.00		1.00		1.00	–	1.00	–
Completely distrust/distrust	1685 (37)	**3.80**	**3.11–4.65**	**4.66**	**3.74–5.80**	**3.26**	**2.74–3.88**	**2.28**	**1.86–2.79**

Bold signals statistically significant p-values (< 0.05).

Odds ratios are the odds of being in the non-adoption of each NPI compared to odds of adoption of that NPI measured with logistic regression.

*CI* confidence interval, *REF* reference group.

### Information and social media platform usage and trust

**Figure 3:**
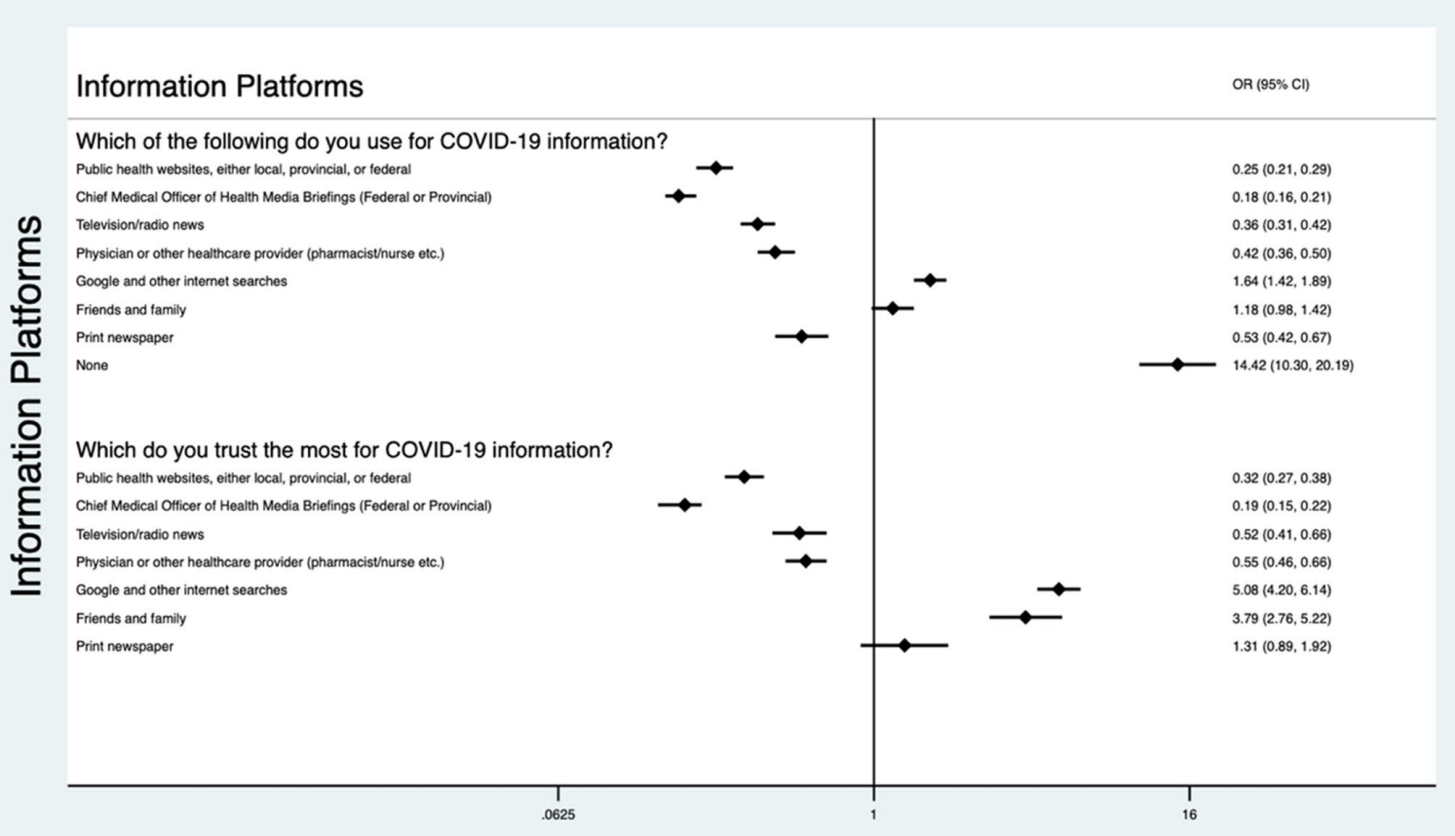
Association between information platforms used and trusted by non-adopters of COVID-19 NPIs compared to adopters. *Participants could pick more than one most trusted source from each list. Odds ratios are the odds of being in the non-adopter cluster compared to odds of being in the adopter cluster.

**Figure 4:**
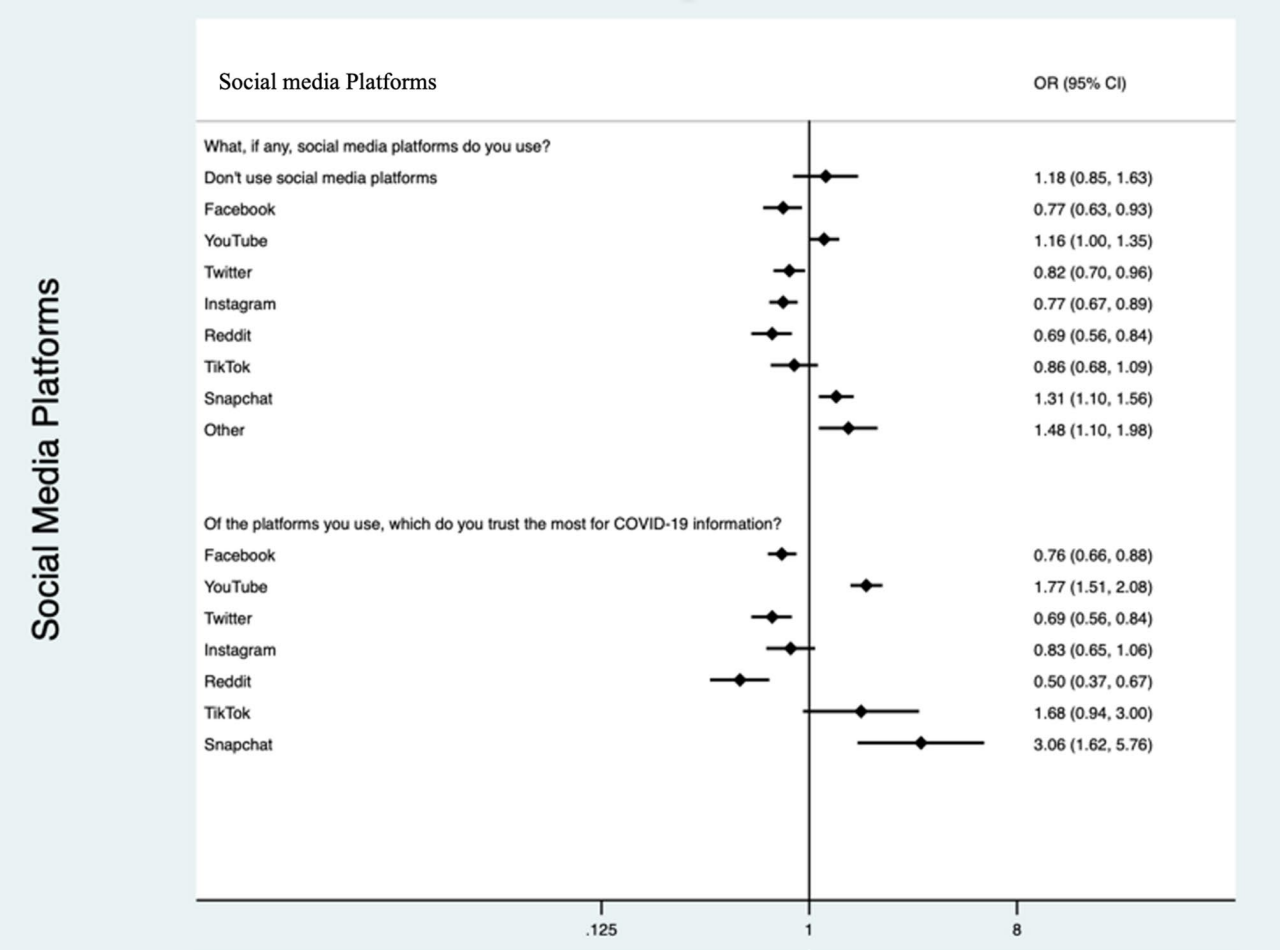
Association between social media platforms used and trusted by non-adopters of COVID-19 NPIs compared to adopters. *Participants could pick more than one most trusted source from each list. Odds ratios are the odds of being in the non-adopter cluster compared to odds of being in the adopter cluster.

The most highly used communication channels and platforms for COVID-19 were public health websites (2897, 64%), health media briefings (2800, 62%), television/radio (2170, 48%) and physician/healthcare provider (1898, 42%). People who reported using or trusting Google or other internet search engines and information from friends and family had higher odds of non-adoption. Whereas those that reported using or trusting public health websites, health media briefings, television/radio or their physician for COVID-19 information were negatively associated with non-adoption (Fig. 3). People who reported that they used none of these sources for COVID-19 information (206, 5%) had higher odds of non-adoption (OR 14.42, 95% CI 10.30–20.19) compared to adopters. Of these individuals who reported they used none of the listed sources for COVID-19 information, 188(91%) use some form of social media and 164 (80%) use Facebook with 85 (41%) reporting trust in Facebook for COVID-19 information. The most common social media platforms used by respondents were Facebook (3842, 85%), YouTube (2919, 65%), Instagram (2287, 51%) and Twitter (1433, 32%). There were 205 (5%) respondents who did not use any form of social media. Respondents who were more likely to use YouTube, Snapchat or a different social media platform than the ones listed had higher odds of non-adoption (Fig. 4).

## Discussion

Based on self-reported adoption of recommended NPIs intended on slowing transmission of COVID-19 including physical distancing, masking in public spaces, avoiding crowded public spaces and staying home when feeling sick, we segmented the population into adopters and non-adopters of NPIs through cluster analysis. Sociodemographic factors associated with non-adoption were: (1) being male, (2) age 18–34 years, (3) Albertans, (4) lower education level, and 5) a higher conservative political leaning. Participants who expressed low concern for COVID-19 had greater odds of being non-adopters. Non-adoption was associated with greater distrust among several institutions including technology, professional services, healthcare and government. Respondents who reported that public health messaging has been unclear and inconsistent and those where messaging has made them less likely to adopt NPIs had greater odds of non-adoption (Table 5).

**Table 5:** Summary of key recommendations to improve NPI adherence.

Key topic	Suggestion
Targeted communication strategies	Communication strategies targeted towards demographics most likely to be non-adopters of NPIs may be most effective, including males, those aged 18–34, individuals with lower education levels and those with a higher conservative political leaning
Increasing individuals concern for COVID-19 infection	Individuals who have little concern regarding COVID-19 infection and disease are less likely to adopt NPIs, therefore messaging that increases concern may be effective
Clarity and consistency of messaging is a requirement to promote adoption	Studies have identified that if messaging is not clear or consistent, individuals have less confidence in that messaging source and are less likely to adhere to the message^52,57,62,63^
Messaging to highlight the effectiveness of NPIs	Individuals in the non-adopter cluster believed that NPIs were not effective in preventing COVID-19 transmission, therefore communication that highlights the efficacy of NPIs may be helpful in promoting their uptake
Improving trust in institutions	Institutions, particularly government and healthcare, need to increase the trust individuals have in them, in order to improve adherence to the messaging they are providing

Consistent with other research, males and young adults in our study reported lower adherence to public health recommendations^20,21,32^. In April 2020, a cross-sectional survey completed in Alberta and Ontario found that the highest non-adoption of NPIs occurred among males, age 16–29 years old, Alberta residents, with low COVID-19 knowledge and low concern^20^. Despite extensive resources and effort put towards messaging campaigns, this non-adopter population appears to be quite similar in characteristics from this earlier survey in April 2020^20^ to our current survey in November 2020. This demonstrates the challenges of changing individuals’ attitudes and behaviours regarding NPIs.

Political leaning was the strongest sociodemographic predictor of adoption. A recent survey found only very small differences between conservative and liberal supporters in Canada and Republican and Democrats in the US in behavioral responses to the pandemic; however, there were greater differences in confidence in governments and concern about COVID-19^33^. Using geotracking data in the US with 15 million smartphones per day, Gollwitzer et al. found that Republican-leaning counties exhibited lower physical distancing than Democratic-leaning counties^34^. In an analysis of tweets, conservatives were more likely than liberals to believe and spread conspiracy theories and misinformation on the COVID-19 pandemic^35^. Outside of the setting of COVID-19, bipartisan support by government leaders has led to less partisan motivated reasoning. This strategy may be also be effective for combatting COVID-19^36,37^.

We found that NPI non-adopters were more likely to have little concern about themselves or their friends and family becoming ill from SARS CoV-2. Non-adopters were also less likely to live with someone high risk of the disease or know someone who had COVID-19. They were also much more likely to have multiple regular interactions with people outside their household. Prior studies have demonstrated that the strongest facilitators for adoption of physical distancing are wanting to protect oneself and others and feeling a responsibility to protect the community. Prior barriers identified included needing to help friends or family members with errands, feeling lonely, and not trusting the messages from the government about the pandemic^32^. Respondents who expressed distrust in government, healthcare, professional services and technology had lower odds of adoption of any of the assessed NPIs. Trust in government institutions and leaders has been shown to be essential if that country chooses to impose restrictions and maintain public support^38^.

The importance of trust is an intrinsic component to address COVID-19. Dating back to the foundations of modern-day communication, the Aristotelian embodiment of the ethos of the information source as a key component of trust offers important perspectives on the substantive cornerstone for public compliance with the recommended actions^39,40^. Trust also is linked to clarity in communication. Trusted, known public health sources with evidence-based, up-to-date, valid information allow everyone to obtain, process, and understand this information in order to make appropriate health decisions. Lessons from previous infectious disease outbreaks and public health emergencies including HIV/AIDS, H1N1, SARS, and MERS highlight the importance of clarity in communication^41^. Trust in government has been correlated with willingness to adopt protective behaviours in the face of other health threats such as the 2009 H1N1 pandemic^42,43^ and the 2014–2016 West African Ebola epidemic^44^.

A previous survey conducted in Canada at the end of April 2020 reported respondents from Ontario and Quebec as having the least amount of trust in the Canadian government^45^; however, in our study we identified highest distrust among Albertans (48%) followed by Saskatchewan and Manitoba (40%). Other studies have also demonstrated that the higher the degree of trust in the health system, the greater the compliance with guidelines^30,46,47^. We identified a high degree of trust in healthcare among our respondents with 75% reporting they either trusted or completely trusted healthcare; however, there were greater odds of non-adoption in people expressing distrust in the healthcare system.

Belief in the efficacy of NPIs has been found to be critical for compliance with NPI recommendations^48^. The greatest proportion of people in the non-adopter cluster reported that they did not believe that the recommendations work as the reason for having not followed the public health guidelines. People who felt that COVID-19 restrictions were harming the economy had lower odds of adoption of NPIs. Prior research has found that there is a positive association between risk perception and economic threat perception, meaning that people that perceive a personal economic threat may as a result be less likely to adhere to guidelines^30,49^.

Individuals have access to a seemingly endless stream of information on COVID-19 through many different informational and social media platforms. One study evaluating the effect of information overload on the intention to self-isolate found that there was a negative impact of information overload on efficacy as information overload often does not allow for accurate understanding and therefore the uncertainty associated lowers efficacy^50^. Social media misinformation and lack of well-designed education programs without community engagement can impact on compliance and acceptability of NPIs^17^. Mobilizing an effective public health response during a pandemic requires clear communication and trust^48,51^. Non-adopters were more likely to report that public health messaging has been unclear and inconsistent. As well, they were more likely to report that messaging has actually made them less likely to adopt NPIs. Our assessment of the trust people have in institutions (Table 4) and the “information sources” (from learned intermediaries (e.g. physicians, health professionals), news readers (celebrities and known people), social media (anonymous sources), and their government offers guidance for future communication and trust building approaches on COVID-19 NPIs and related efforts with vaccination^52^.

The majority of respondents reported using public health websites, medical officer of health media briefings or TV to get their COVID-19 information; however, non-adopters were more likely to pick none of the provided options, meaning that there was possibly an information platform that we had missed listing or these individuals do not seek out COVID-19 information. Interestingly when respondents were asked if messaging or advertising had an impact on their likelihood to adopt NPIs, those who responded that they had not seen any messaging or advertising had much greater odds of non-adoption. Therefore, it is possible that there are a subgroup of people whom the public health messaging is not reaching, and these people are also more likely to be non-adopters. Future work is needed to identify effective methods and channels of messaging to this group of individuals as there is a shortage of qualitative research addressing this subject.

Facebook, YouTube and Instagram were the most commonly used social media platforms. We found that individuals using YouTube and Snapchat for COVID-19 information had higher odds of non-adoption. Several prior studies have found a negative association between COVID-19 conspiracy beliefs, usage and trust in social media networks with adoption of NPIs^53,54^. One study evaluating conspiracy beliefs associated with COVID-19 on social media found that YouTube had the strongest association with conspiracy beliefs, followed by Facebook^53^. This suggests that targeting these social media platforms may be an effective option for distributing targeted messaging to promote uptake of NPIs for COVID-19 prevention. Overall, effective, credible, consistent and culturally informed health communication is vital in influencing positive health behaviours and building trust^41,55,56^, especially in terms of encouraging people to adhere to COVID-19 control measures and NPIs.

There are several strengths and limitations to our work. This was a cross-sectional survey representing persons attitudes and behaviors at the time of this study, which will continue to change over time as the pandemic evolves. The survey recruited participants from an existing voluntary nationwide panel designed to be representative of the Canadian population; however, by using a panel there will be a component of selection bias as participants have volunteered to partake in research surveys and have access to electronic devices to do so. A non-response bias is possible as 1395 participants began but never fully completed the survey and were therefore excluded, however based on age, sex, ethnicity, education level and province of residence, these data were missing at random. Despite having a large sample size and being conducted in both English and French, generalizability may be limited. It is possible that some of the questions in the survey may have been interpreted differently by participants leading to variability in responses. It is also possible that individuals’ motivations towards non-adoption of public health behaviours are complex and intertwined and not easily characterized or captured by survey tools. Future work including repeat surveys and qualitative studies to assess public attitudes and behaviors through this changing pandemic will be key in maintaining effective messaging promoting adoption of NPIs.

Throughout the vaccination roll-out, adherence to and adoption of NPIs to reduce the spread of COVID-19 will remain critical. NPIs are most successful when there is a greater uptake from the public. This work provides a significant contribution to the COVID-19 literature through characterization of individuals more likely to be non-adopters of NPIs. An in-depth review of these individuals’ sociodemographic factors, behaviours and attitudes towards COVID-19 and the barriers for NPI adoption is presented. We deliver a unique perspective though a Canadian national survey at a critical time during the pandemic, just at the initiation of the second wave with rising COVID-19 cases occurring across the country. This information will be useful for developing effective communication strategies including; knowledge translation tools, marketing programs and community engagement, targeted toward non-adopters of NPIs, in both message content and effective platforms for dissemination.

## Methods

### Study design

A cross-sectional survey (Supplementary Fig. S1) was used to assess respondents’ self-reported adherence to NPIs including physical distancing, masking, avoiding public spaces and staying home when sick as methods to reduce spread of COVID-19. This survey was informed by an online survey and focus groups conducted in Alberta, Canada by the research team^57,58^. Information on sociodemographic factors, attitudes towards COVID-19 and NPIs as well as risk and trust measures were collected. Participants were also asked about their usage and trust in information and social media platforms for COVID-19 information. This study was approved by the University of Calgary Conjoint Health Research Ethics Board (REB20-1228). Informed consent was obtained, and participation was voluntary. The Strengthening the Reporting of Observational Studies in Epidemiology (STROBE) checklist was used to report our findings^59^.

### Participants and setting

The Angus Reid Forum was used for selection of participants^31^. Eligibility was defined as: (1) aged 18 years or older, (2) live in a Canadian province, (3) speak either English or French, and (4) have access to the internet. The survey was administered between October 27 and November 2, 2020, in both French and English by the Angus Reid Institute, a national, not-for-profit, research foundation. The survey was programed using Askia^60^ and launched using Platform One^61^. The survey was distributed to 14,887 potential participants who were randomly selected from the Angus Reid Forum of 70,000 individuals who are representative of the Canadian population^31^, in order to obtain a sample size of 4500. Sample size calculated for a 95% confidence interval with a margin of error of 3% for the adult population of Canada was 1068. However, in order to be able to stratify for equal representation of Alberta residents and residents of the other Canadian provinces combined we used a sample size of 4500 respondents. This sampling strategy was used to allow for comparison of two Canadian applications used to facilitate contact tracing, ABTraceTogether (a contact tracing application which is only available in Alberta) and COVID Alert (an exposure notification application available in eight provinces and the Northwest Territories).

### Variables and measurement

The main outcome measure was adoption of NPIs assessed by respondents answering the question; over the last few weeks, how often have you been performing each of the behaviors: (1) social/physical distancing-keeping at least 2 m from other people who are not in your social bubble, (2) wearing a mask in public indoor spaces when physical distancing is not possible, (3) avoiding places & activities where you would interact with a large number of people outside your household, and (4) staying home if you were sick with any symptoms, even mild ones. Adoption of these NPIs was assessed on a Likert scale of; all the time, most of the time, sometimes, rarely and never.

Sociodemographic factors including sex, age, province of residence, household income, highest level of education, ethnicity, and political leaning were collected and categorized. Questions identifying attitudes towards COVID-19, public heath recommendations and reasons for non-adoption of recommendations were asked. Likert scales were used to assess agreement with statements on public health messaging and restrictions, how effective people believe public health recommendations are at reducing spread of COVID-19, effectiveness of public messaging thus far, and trust in specific institutions. Respondents’ usage and trust of information platforms and social media platforms were collected.

### Statistical analysis

Descriptive statistics (percentage frequencies) were calculated for all sociodemographic characteristics, attitudes toward COVID-19 and towards NPIs and adoption of NPIs. Categorical variables were compared using chi-squared tests. Respondents who had not completed all survey responses were excluded, therefore there were no missing data.

Cluster analysis was used as a data-driven method to identify the most important and meaningful patterns in the survey. This analysis highlighted how different or similar individuals are in their attitudes toward NPIs such that empirical patterns can be summarized in an insightful and concise manner. The goal of cluster analysis is to estimate a limited number of clusters with the most similarity within clusters but most dissimilarity between clusters. Clustering was based on individuals’ attitudes and behaviors toward NPIs (limiting social gatherings, physical distancing, using face masks, avoiding public spaces and quarantining when sick), this includes reported actions and opinions on effectiveness of NPIs and on clarity of public messaging about NPIs.

A series of statistical tests were conducted to determine the clustering method and the optimal number of clusters to explain the empirical variations in the data. The Kmeans algorithm was used for cluster analysis to partition the dataset into two distinct non-overlapping clusters labeled as adopter and non-adopter clusters. Kmeans is an iterative algorithm that assigns observations or data points to a cluster with the objective to minimize the sum of squared distance between the data points and the cluster’s arithmetic mean of all the data points that belong to that cluster. The output of analysis in this part is a data-driven and detailed way to allocate each observation to their appropriate cluster and generate a clustering indicator variable (adopter and non-adopter cluster) to be used for further investigation.

Logistic regression was used to calculate the odds ratio (OR) with 95% confidence interval (CI) for the non-adoption cluster compared to the adoption cluster as a reference. Logistic regression was used to identify risk factors for non-adoption of NPIs by sociodemographic factors, attitudes towards COVID-19, attitudes towards NPIs and towards public health messaging, and trust in specific institutions. Logistic regression was also used to identify communication channels and social media platforms at higher odds of being used and trusted by non-adopters. Backwards stepwise regression identified sociodemographic factors significantly associated with adoption clusters. These sociodemographic factors were included in multivariable regression models estimating the adjusted odds ratio (aOR) comparing adoption clusters with (1) attitudes towards COVID-19, (2) attitudes towards NPIs and (3) attitudes toward public health messaging. All P-values were two-tailed tests, and the statistical significance level was set at P < 0.05. All statistical analyses were performed using STATA version 15.1 (College Station, TX).

### Declarations

All experiments were performed in accordance with relevant guidelines and regulations.

### Ethics approval

The study was approved by the University of Calgary Conjoint Health Research Ethics Board (REB20-1228).

### Consent for publication

All authors give their consent to publication of this work.

## Supplementary Information

Supplementary Information.

## Data Availability

The summary dataset used and or analyzed during the current study are available from the corresponding author on a reasonable request.
