# Supplementary material for: Characterization of non-adopters of COVID-19 non-pharmaceutical interventions through a national cross-sectional survey to assess attitudes and behaviours
Source: Sci Rep. 2021 Nov 5;11:21751. doi: 10.1038/s41598-021-01279-2 (PMC8571421; doi:10.1038/s41598-021-01279-2)
Supplement: Supplementary file 1 — Supplementary Information. [file 41598_2021_1279_MOESM1_ESM.docx]

Characterization of non-adopters of COVID-19 non-pharmaceutical interventions: a national cross-sectional survey to assess attitudes and behaviours

*Raynell Lang MD^1^, Omid Atabati MSc^2^, Robert J. Oxoby PhD^2^, Mehdi Mourali PhD^3^,

Blake Shaffer PhD^2^, Hasan Sheikh MD MPA^4^, Madison M. Fullerton MSc^5^,

Theresa Tang BHSc(Hons)^5^, Jeanna Parsons Leigh PhD^6^, Braden J. Manns MD MSc^1,5^, Deborah A. Marshall PhD^1,5^, Noah M. Ivers MD PhD^7^, Scott C. Ratzan MD MPA MA^8^,

Jia Hu MD^5^, Jamie L. Benham MD PhD^1,5^

^1^ Department of Medicine, Cumming School of Medicine, University of Calgary, Calgary, AB, Canada

^2^ Department of Economics, Faculty of Arts, University of Calgary, Calgary, AB, Canada

^3^ Haskayne School of Business, University of Calgary, Calgary, AB, Canada

^4^ Department of Family and Community Medicine, University of Toronto, Toronto, ON, Canada

^5^ Department of Community Health Sciences, Cumming School of Medicine, University of Calgary, Calgary, AB, Canada

^6^ Faculty of Health, School of Health Administration, Dalhousie University, Halifax, Nova Scotia, Canada, Department of Critical Care Medicine, University of Calgary, Calgary, Alberta, Canada

^7^ Women's College Hospital Institute for Health System Solutions and Virtual Care, Women's College Hospital, Toronto, ON, Canada; Institute for Health Policy, Management and Evaluation, University of Toronto, Toronto, ON, Canada

^8^ City University of New York Graduate School of Public Health & Health Policy, New York, NY, USA

| **Supplemental Table 1. Participant characteristics associated with adoption of COVID-19 non-pharmaceutical interventions** | | | | | | | | | | |
| --- | --- | --- | --- | --- | --- | --- | --- | --- | --- | --- |
| **Characteristic** | **Total**  **N=4503(%)** | **Physical Distancing** | | **Masking** | | | **Avoiding Crowded Spaces** | | **Staying home when sick** | |
|  |  | **Always/**  **Mostly**  **(N=3782)(%)** | **Sometimes/**  **Rarely/Never**  **(N=721)(%)** | **Always/**  **Mostly (N=3873)(%)** | **Sometimes/**  **Rarely/Never**  **(N=631)(%)** | | **Always/**  **Mostly (N=3517)(%)** | **Sometimes/**  **Rarely/Never**  **(N=986)(%)** | **Always/**  **Mostly (N=3857)(%)** | **Sometimes/**  **Rarely/Never**  **(N=646)(%)** |
| **Biologic Sex** |  |  |  |  | |  |  |  |  |  |
| Male | 2205 (49) | 1756 (80) | 449 (20) | 1798 (82) | 407 (18) | | 1598 (72) | 607 (28) | 1780 (81) | 425 (19) |
| Female | 2298 (51) | 2026 (88) | 272 (12) | 2075 (90) | 223 (10) | | 1919 (84) | 379 (16) | 2077 (90) | 221 (10) |
| **Age (years)** |  |  |  |  |  | |  |  |  |  |
| 18-34 | 1341 (30) | 1079 (80) | 262 (20) | 1173 (87) | 168 (13) | | 1003 (75) | 338 (25) | 1143 (85) | 198 (15) |
| 35-54 | 1589 (35) | 1279 (80) | 310 (20) | 1324 (83) | 265 (17) | | 1197 (75) | 392 (25) | 1338 (84) | 251 (16) |
| $\geq$55 | 1573 (35) | 1424 (91) | 149 (9) | 1376 (87) | 197 (13) | | 1317 (84) | 256 (16) | 1376 (87) | 197 (13) |
| **Province** |  |  |  |  | |  |  |  |  |  |
| Alberta | 2003 (44) | 1589 (79) | 414 (21) | 1590 (79) | 413 (21) | | 1426 (71) | 577 (29) | 1667 (83) | 336 (17) |
| British Columbia | 502 (11) | 446 (88.8) | 56 (11) | 436 (87) | 66 (12) | | 426 (85) | 76 (15) | 436 (87) | 66 (13) |
| Prairie Provinces^+^ | 445 (10) | 365 (82) | 80 (18) | 360 (81) | 85 (19) | | 352 (79) | 93 (21) | 378 (85) | 67 (15) |
| Ontario | 800 (18) | 706 (88) | 94 (12) | 763 (95) | 37 (5) | | 689 (86) | 111 (14) | 713 (89) | 87 (11) |
| Quebec | 502 (11) | 454 (90) | 48 (10) | 489 (97) | 37 (3) | | 425 (85) | 77 (15) | 441 (88) | 61 (12) |
| Atlantic Provinces^+^ | 251 (6) | 222 (88) | 29 (12) | 235 (94) | 16 (6) | | 199 (79) | 52 (21) | 222 (88) | 29 (12) |
| **Annual Household Income** |  |  |  |  |  | |  |  |  |  |
| <$50,000 | 951 (21) | 832 (87) | 119 (13) | 857 (90) | 94 (10) | | 777 (82) | 174 (18) | 826 (87) | 125 (13) |
| $50,000-$99,999 | 1332 (30) | 1130 (85) | 202 (15) | 1151 (86) | 181 (14) | | 1048 (79) | 284 (21) | 1132 (85) | 200 (15) |
| $100,000-$199,999 | 1354 (30) | 1121 (83) | 233 (17) | 1148 (85) | 206 (15) | | 1042 (77) | 312 (23) | 1161 (86) | 193 (14) |
| $\geq$$200,000 | 214 (5) | 163 (76) | 51 (24) | 172 (80) | 42 (20) | | 144 (67) | 70 (33) | 175 (82) | 39 (18) |
| Rather Not Say | 652 (15) | 536 (82) | 116 (18) | 545 (84) | 107 (16) | | 506 (78) | 146 (22) | 563 (86) | 89 (14) |
| **Highest Education** |  |  |  |  |  | |  |  |  |  |
| High School Graduate or less | 898 (20) | 731 (81) | 167 (19) | 731 (81) | 167 (19) | | 682 (76) | 216 (24) | 749 (83) | 149 (17) |
| Some College or Trade School | 840 (19) | 694 (83) | 146 (17) | 692 (82) | 148 (18) | | 640 (76) | 200 (24) | 708 (84) | 132 (16) |
| College or Trade School | 997 (22) | 816 (82) | 181 (18) | 836 (84) | 161 (16) | | 766 (77) | 231 (23) | 828 (83) | 169 (17) |
| Some University | 454 (10) | 382 (84) | 72 (16) | 392 (86) | 62 (14) | | 351 (77) | 103 (23) | 392 (86) | 62 (14) |
| University Degree | 1314 (29) | 1159 (88) | 155 (12) | 1222 (93) | 92 (7) | | 1078 (82) | 236 (19) | 1180 (90) | 134 (10) |
| **Race/Ethnicity** |  |  |  |  | |  |  |  |  |  |
| Caucasian | 3866 (85) | 3253 (84) | 613 (16) | 3326 (86) | 540 (14) | | 3007 (78) | 859 (22) | 3310 (86) | 556 (14) |
| Indigenous/First Nations/Metis/Inuit | 228 (5) | 186 (82) | 42 (18) | 187 (82) | 41 (18) | | 173 (76) | 55 (24) | 199 (87) | 29 (13) |
| Chinese/Filipino/Other Asian | 124 (3) | 107 (86) | 17 (14) | 114 (92) | 10 (8) | | 112 (90) | 12 (10) | 109 (88) | 15 (12) |
| Caribbean/South American/African | 70 (2) | 56 (80) | 14 (20) | 64 (91) | 6 (9) | | 56 (80) | 14 (20) | 58 (83) | 12 (17) |
| Middle Eastern/Central Asian/South Asian | 69 (2) | 61 (88) | 8 (12) | 64 (93) | 5 (7) | | 59 (86) | 10 (14) | 61 (88) | 8 (12) |
| Other | 146 (3) | 119 (82) | 27 (18) | 118 (81) | 28 (19) | | 110 (75) | 36 (35) | 120 (82) | 26 (18) |
| **Political Leaning** |  |  |  |  |  | |  |  |  |  |
| Very Liberal | 603 (13) | 562 (93) | 41 (7) | 591 (98) | 12 (2) | | 547 (91) | 56 (9) | 559 (93) | 44 (7) |
| Liberal | 805 (18) | 749 (93) | 56 (7) | 782 (97) | 23 (3) | | 715 (89) | 90 (11) | 734 (91) | 71 (9) |
| Slightly liberal | 433 (10) | 400 (92) | 33 (8) | 417 (96) | 16 (4) | | 376 (87) | 57 (13) | 385 (89) | 48 (11) |
| Moderate/middle of the road | 1029 (23) | 908 (88) | 121 (12) | 937 (91) | 92 (9) | | 855 (83) | 174 (17) | 915 (89) | 114 (11) |
| Slightly Conservative | 485 (11) | 378 (78) | 107 (22) | 389 (80) | 96 (20) | | 332 (68) | 153 (32) | 393 (81) | 92 (19) |
| Conservative | 807 (18) | 575 (71) | 232 (29) | 566 (70) | 241 (30) | | 508 (63) | 299 (37) | 625 (77) | 182 (23) |
| Very Conservative | 336 (7) | 205 (61) | 131 (39) | 186 (55) | 150 (45) | | 180 (54) | 156 (46) | 241 (72) | 95 (28) |

^+^Prairie provinces included Saskatchewan and Manitoba; Atlantic provinces include Nova Scotia, New Brunswick, Prince Edward Island and Newfoundland and Labrador

| **Supplemental Table 2:** | | | |
| --- | --- | --- | --- |
| **Non-pharmaceutical intervention** | **Non-Adopter Cluster** | **Adopter Cluster** | **Total** |
|  | **(N=994)**  Mean (SD) | **(N=3504)**  Mean (SD) | **(N=4498)**  Mean (SD) |
| **Over the past few weeks, how often have you been doing each of the following**  (1=All the time, 2= Most of the time, 3= Sometimes, 4= Rarely, 5= Never) | | | |
| **Physical distancing** | 2.89 (± 1.04) | 1.55 (± 0.60) | 1.83 (± 0.89) |
| **Masking** | 2.78 (± 1.44) | 1.19 (± 0.50) | 1.54 (± 1.04) |
| **Avoiding public places** | 3.34 (± 1.27) | 1.47 (± 0.69) | 1.88 (± 1.16) |
| **Staying home when sick** | 2.65 (± 1.52) | 1.30 (± 0.77) | 1.60 (± 0.1.14) |
